# Musculoskeletal pain in university students: lifestyle determinants of prevalence and burden

**DOI:** 10.3389/fpubh.2026.1831482

**Published:** 2026-07-10

**Authors:** Yan Li, Juan Liang, Ji Ma, Zhenchao Jia, Xiaomeng Li, Qiaoli Su

**Affiliations:** 1 Sichuan University Hospital, Sichuan University, Chengdu, China; 2 School of Public Health, Zhengzhou University, Zhengzhou, China; 3 West China School of Public Health and West China Fourth Hospital, Sichuan University, Chengdu, China; 4 General Practice Ward/International Medical Center Ward, General Practice Medical Center, National Clinical Research Center for Geriatrics, West China Hospital, Sichuan University, Chengdu, China

**Keywords:** lifestyle behaviors, musculoskeletal pain, pain burden, pain prevalence, public health interventions, university students

## Abstract

**Background:**

Musculoskeletal pain (MSP) is a leading cause of disability worldwide, yet evidence among young adults remains limited. University students face unique lifestyle risks, including prolonged sedentary study, poor sleep, and excessive screen use. This study aimed to estimate MSP prevalence and burden, compare lifestyle behaviors between students with and without pain, and identify independent predictors of pain occurrence and severity.

**Methods:**

A cross-sectional web-based survey was conducted among students at Sichuan University, China. A structured lifestyle questionnaire assessed physical activity, diet, sedentary behavior, sleep, screen use, and pain burden across major body sites. Univariate and multivariate logistic regression were used to identified risk factors of MSP prevalence and pain burden.

**Results:**

A total of 9,122 students participated (mean age 21.1 ± 1.7 years; 44.5% female). MSP prevalence was 58.7%, with the neck (43.6%), back (37.1%), and shoulders (36.2%) most affected. Pain burden was considerable, with 22.6% reporting moderate and 18.5% severe pain. Multivariable analysis showed that female gender (*OR* = 1.99, 95% CI: 1.80–2.19), poor sleep (*OR* = 2.81, 95% CI: 2.26–3.49), poor diet (*OR* = 1.83, 95% CI: 1.59–2.10), high sedentary risk (*OR* = 1.33, 95% CI: 1.10–1.60), long screen duration (*OR* = 1.49, 95% CI: 1.31–1.69), and low physical activity (*OR* = 1.14, 95% CI: 1.02–1.27) were independent predictors of MSP prevalence. For pain burden, poor sleep (*OR* = 3.72, 95% CI: 2.86–4.83), poor diet (*OR* = 1.90, 95% CI: 1.66–2.18), high sedentary risk (*OR* = 1.68, 95% CI: 1.42–1.99), long screen duration (*OR* = 1.37, 95% CI: 1.20–1.55), low physical activity (*OR* = 1.04, 95% CI: 1.02–1.15), and female gender (*OR* = 1.90, 95% CI: 1.73–2.08) were the strongest predictors.

**Conclusion:**

MSP is highly prevalent among university students, with a substantial proportion experiencing moderate to severe burden. Lifestyle factors influence both pain occurrence and severity. Poor sleep, poor diet, sedentary behavior, long screen use and insufficient physical activity were consistently associated with higher risk. These findings highlight the need for multidomain but tailored interventions to reduce both prevalence and burden, supporting healthier long-term habits and effective campus health strategies.

## 1 Introduction

Musculoskeletal pain (MSP) is one of the most prevalent health problems worldwide, exerting a substantial impact on quality of life and contributing to considerable economic burden (1). MSP encompasses degenerative and inflammatory conditions affecting muscles, tendons, ligaments, joints, and associated neurological structures, with symptoms ranging from mild discomfort to severe disability (2). These disorders interfere with daily activities such as walking, sitting, or studying, and their burden has risen markedly in recent decades. According to the Global Burden of Disease (GBD) study, disability-adjusted life years attributable to MSP (excluding low back pain) increased by more than 30% between 1990 and 2019, while low back pain consistently ranks among the leading causes of years lived with disability (3).

Although MSP is often associated with aging and occupational exposures, evidence indicates that its prevalence is also increasing among young adults. Studies from diverse settings report prevalence rates of 50%−70% in undergraduate populations, particularly among students facing high academic demands (4–6). University students are vulnerable due to prolonged sitting, inadequate postures during study or laboratory work, and elevated stress levels, which may exacerbate musculoskeletal symptoms (7, 8). Despite this, research on MSP in younger adults remains limited, as chronic pain has traditionally been studied in older populations.

Musculoskeletal pain is influenced by a complex interplay of internal and external factors. Psychological stress, anxiety, and depression have been shown to amplify pain perception, while physiological mechanisms such as hormonal changes and inflammatory processes contribute to vulnerability (9). External determinants, including ergonomic study environments, socioeconomic status, and academic workload, further shape pain risk (10). These broader influences highlight the multifactorial nature of MSP. Within this context, lifestyle behaviors are increasingly recognized as important determinants. Physical inactivity, sedentary behavior, poor diet, inadequate sleep, and excessive screen use have all been implicated in musculoskeletal disorders (11–16). Sedentary behavior, defined as waking time spent sitting or reclining with low energy expenditure, is associated with adverse outcomes including cardiovascular disease, metabolic disorders, and MSP (13, 17). Similarly, prolonged screen use and poor ergonomics contribute to neck and shoulder pain, while unhealthy dietary patterns and poor sleep quality may exacerbate systemic inflammation and pain sensitivity (14, 16). Conversely, healthy lifestyle behaviors such as regular physical activity and balanced diet are protective, yet evidence on their role in young adults remains inconsistent (18).

Despite growing recognition of these associations, few studies have comprehensively examined the interaction between multiple lifestyle domains and pain burden in large university cohorts. Moreover, evidence from Chinese students is scarce, despite the unique academic pressures and lifestyle transitions they face. To address these gaps, we conducted a large cross-sectional study among students at Sichuan University, Chengdu, China. As the largest comprehensive university in the southwest of China, the university enrolls students from across the country, with over 20,000 new students admitted each academic year. Using a structured lifestyle questionnaire adapted from validated international instruments, we assessed physical activity, diet, sedentary behavior, sleep, screen use, and pain burden. Our objectives were: (1) to estimate the prevalence and severity of MSP among university students; (2) to compare lifestyle behaviors between students with and without pain; and (3) to identify independent lifestyle risk factors of pain occurrence and pain severity. This study provides novel insights into behavioral determinants of pain in young adults and informs targeted campus health interventions.

## 2 Methods

### 2.1 Study participants and design

This study was approved by the Ethics Review Committee of the West China School of Public Health and West China Fourth Hospital, Sichuan University (Approval No. Gwll2024060). Participants were recruited from Sichuan University, Chengdu, China—the largest comprehensive university in Sichuan Province. Although located in southwest China, the university enrolls students from across the country, with over 20,000 new students admitted each academic year.

A cross-sectional design was adopted using a web-based survey. Investigators explained the study objectives, and informed consent was obtained prior to participation. Students completed the questionnaire electronically via an online platform (http://www.wjx.cn). Participation was voluntary, responses were anonymized, and confidentiality was assured. Exclusion criteria included: (1) history of mental or psychological disorders, and (2) history of hypertension, diabetes, or other congenital diseases.

The sample size was calculated based on a response rate of 30%, a 99% confidence interval, a 5% margin of error, and a total student population of 27,000. The minimum required sample size was 2,160; however, the final dataset included 9,122 students. This oversampling reflects both the high response rate achieved and our aim to maximize representativeness across diverse student subgroups. Including a substantially larger sample size enhances the precision of prevalence estimates and strengthens the robustness of both univariate and multivariable analyses. It also allows for more reliable subgroup comparisons (e.g., by gender or lifestyle domains) and reduces the margin of error in effect size estimates. At the same time, oversampling increases sensitivity to detect small differences that may reach statistical significance but have limited clinical or public health relevance. To address this, interpretation of results emphasizes effect sizes and practical significance rather than statistical significance alone.

### 2.2 Measures

A structured Lifestyle Questionnaire was developed to assess health-related behaviors and pain burden. The instrument was informed by international frameworks and validated tools, and comprised six domains: demographic information, physical activity, dietary habits, sedentary behavior, sleep behavior, screen use, and pain assessment.

#### 2.2.1 Demographics and anthropometrics

Age, sex, ethnicity, academic discipline, grade level, height, and weight were collected. Body Mass Index (BMI) was calculated from self-reported height and weight.

#### 2.2.2 Physical activity

Assessed using the International Physical Activity Questionnaire–Short Form (IPAQ-SF) (19). Weekly metabolic equivalent (MET) minutes were calculated by summing walking (3.3 MET × minutes × days), moderate activity (4.0 MET × minutes × days), and vigorous activity (8.0 MET × minutes × days). Based on WHO recommendations, activity levels were classified as high (≥3,000 MET min/week), moderate (600–2,999 MET min/week), or low (< 600 MET min/week) (20). For scoring, high activity was assigned 4 points. Moderate activity was scored as 3 points for participants reporting 1,200–2,999 MET min/week and 2 points for participants reporting 600–1,199 MET min/week. Low activity was scored as 1 point for participants reporting 180–599 MET min/week and 0 for participants reporting < 180 MET min/week.

#### 2.2.3 Dietary behavior

Items were adapted from the Chinese Dietary Guidelines (2022) and international food frequency questionnaires (21, 22). Indicators included meal regularity, intake of whole grains, fruits, vegetables, dairy, protein sources, and consumption of sugary, fried, or processed foods, late-night meals, restaurant dining, take-out food, and sugar-sweetened beverages. Frequency was categorized as < 1, 1–2, 3–4, 5–6, or 7 times/week. Responses were scored on a 0–2 scale, with higher scores indicating healthier patterns. Participants reporting ≥5 times/week scored 2 points, 3–4 times/week scored 1 point, and < 3 times/week scored 0. The total dietary score was classified as good (≥15), moderate (10–14), or poor ( ≤ 9).

#### 2.2.4 Sedentary behavior

Sedentary behavior was evaluated using items adapted from the IPAQ and Sedentary Behavior Questionnaire (SBQ) (19, 23). Indicators included daily sitting time (< 4, 4–6, 6–8, 8–10, >10 h/day) and continuous sitting duration (< 30, 30–60, 60–120, 120–180, >180 min). Sitting < 4 h/day was scored 4 points, 4–6 h/day 3 points, 6–8 h/day 2 points, 8–10 h/day 1 point, and >10 h/day 0 points. Continuous sitting < 30 min was scored 4 points, 30–60 min 3 points, 60–120 min 2 points, 120–180 min 1 point, and >180 min 0 points. Scores were summed and classified as low risk (≥6 points), moderate risk (3–5), or high risk ( ≤ 2).

#### 2.2.5 Sleep behavior

Assessed using five items adapted from the Pittsburgh Sleep Quality Index (PSQI), including bedtime (before 23:00, 23:00–0:00, 0:00–1:00, 1:00–2:00, after 2:00), frequency of sleep before 23:00 (< 1, 1–2, 3–4, 5–6, or 7 days/week), sleep duration (< 5, 5–6, 6–7, 7–8 or >8 h per day), sleep latency (< 5, 5–15, 16–30, 31–60 or >60 min), subjective sleep quality (very bad, bad, medium, good, very good), and daytime sleepiness (never, < 1, 1–2, 3–5, or >5 days/week) (24). Each item was scored 0–4, yielding a total score of 0–20. Sleep quality was classified as good (≥16), moderate (11–15), or poor ( ≤ 10).

#### 2.2.6 Screen use

Measured by daily screen time across devices (computers, smartphones, tablets) (14, 25). Screen use duration < 1 h was scored 4 points, 1–3 h was scored 3 points, 3–6 was scored 2 points, 6–9 h was scored 1 point and >9 h was scored 0. Screen use was classified as short ( ≤ 3 h/day), moderate (3–6 h/day), or long duration (≥6 h/day).

#### 2.2.7 Pain assessment

Pain was assessed across six major body regions (head, neck, shoulders, back, waist, hips/thighs). Other sites (elbows, wrists/hands, knees, ankles/feet) were recorded but not scored. Pain severity was rated on a 5-point scale (0 = no pain to 4 = very severe). Pain burden was classified as mild ( ≤ 2 sites, all mild or below), moderate (≥3 sites or ≥1 moderate site), or severe (≥5 sites or ≥1 severe site) for all participants. For descriptive comparisons, pain burden was dichotomized into “with pain” (any level) and “without pain” (none).

The questionnaire was content-validated by experts in clinical medicine, public health, and medical education. A pilot test with 50 students confirmed comprehension and ease of completion. Reliability and validity testing showed acceptable values: physical activity (Cronbach's α = 0.759, KMO = 0.658), diet (α = 0.751, KMO = 0.652), sleep (α = 0.707, KMO = 0.649), sedentary/screen use (α = 0.782, KMO = 0.701), and pain assessment (α = 0.748 KMO = 0.622).

### 2.3 Statistical analysis

Continuous variables (e.g., age, BMI, scores) are presented as mean ± standard deviation. Categorical variables (e.g., sex, major, academic year) are presented as frequencies. Independent sample *t*-tests compared continuous variables between pain and no-pain groups. Demographic comparisons between students with and without musculoskeletal pain were conducted to explore subgroup differences and to identify potential confounders for regression analyses, which ensure that baseline characteristics were examined prior to multivariable modeling. Chi-square tests assessed differences in categorical variables (sex, major, academic year, MSP prevalence). For multiple comparisons of prevalence across academic grades, chi-square tests with Bonferroni correction were applied.

Univariable logistic regression was first performed to examine crude associations between demographic and lifestyle factors and musculoskeletal pain (MSP) prevalence. Variables showing statistical significance (*p* < 0.05) and/or clinical or public health relevance were then entered into multivariable logistic regression models to identify independent predictors of MSP prevalence. Odds ratios (ORs) with 95% confidence intervals (CIs) are reported as measures of effect size.

Pain burden, classified as mild, moderate, or severe, was analyzed using multivariable ordinal logistic regression. This approach allowed estimation of the likelihood of reporting higher burden categories while adjusting for potential confounders. ORs with 95% CIs are reported for each predictor relative to the reference group. Model fit was assessed using the Hosmer–Lemeshow goodness-of-fit test for logistic models.

All analyses were conducted using SPSS version 26 (IBM Corporation). Statistical significance was set at *p* < 0.05, and effect sizes (ORs) are presented to convey the magnitude of associations.

## 3 Results

### 3.1 Demographic information and MSP prevalence

A total of 9,122 complete responses were analyzed. The mean age of participants was 21.1 ± 1.7 years (range: 16–24), with 5,063 males (55.5%) and 4,059 females (44.5%). The mean BMI was 21.1 ± 2.6. Participant characteristics are summarized in Table 1, which presents unadjusted comparisons between students with and without MSP.

**Table 1:** Demographic characteristics lifestyle behavior of participants (*N* = 9,122).

Variables	Total (9,122)	No pain (*n* = 3,767)	Pain (*n* = 5,355)	*p*-value
Age (mean ± SD)	20.553 ± 0.90	20.343 ± 0.85	20.703 ± 0.92	< 0.001^*^
Gender				< 0.001^*^
Female	4,059 (44.5)	1,308 (32.2)	2,751 (67.8)	
Male	5,063 (55.5)	2,459 (48.6)	2,604 (51.4)	
BMI (mean ± SD)		21.683 ± 0.38	21.443 ± 0.32	0.02^*^
Grade				< 0.001^*^
Undergraduates	5,489 (60.1)	2,422 (44.1)	3,067 (55.9)	
Master candidates	2,860 (31.4)	1,073 (37.5)	1,787 (62.5)	
Ph.D candidates	773 (8.5)	272 (35.2)	501 (64.8)	
Major				< 0.001^*^
Humanity	1,979 (21.7)	725 (36.6)	1,254 (63.4)	
Science	1,659 (18.2)	731 (44.1)	928 (55.9)	
Engineer	3,828 (42.0)	1,671 (43.7)	2,157 (56.3)	
Medicine	980 (10.7)	392 (40.0)	588 (60.0)	
Others	676 (7.4)	248 (36.6)	428 (63.4)	
Lifestyle				
Physical activity				< 0.001^*^
Low	3,051 (33.4)	1,133 (37.1)	1,918 (62.9)	
Moderate	2,507 (27.5)	979 (39.1)	1,528 (60.9)	
High	3,564 (39.1)	1,655 (46.4)	1,909 (53.6)	
Diet				< 0.001^*^
Poor	2,315 (25.4)	763 (33.0)	1,552 (67.0)	
Moderate	5,046 (55.3)	2,057 (40.8)	2,989 (59.2)	
Good	1,761 (19.3)	947 (53.8)	814 (46.2)	
Sedentary behavior				< 0.001^*^
High risk	691 (7.6)	231 (33.4)	460 (66.6)	
Moderate risk	5,328 (58.4)	2,060 (38.7)	3,268 (61.3)	
Low risk	3,103 (34.0)	1,476 (47.6)	1,627 (52.4)	
Sleep				< 0.001^*^
Poor	3,789 (41.5)	1,261 (33.3)	2,528 (66.7)	
Moderate	4,870 (53.4)	2,196 (45.1)	2,674 (54.9)	
Good	463 (5.1)	310 (67.0)	153 (33.0)	
Screen use				< 0.001^*^
Long duration	3,167 (34.7)	1,085 (34.3)	2,082 (65.7)	
Moderate duration	4,042 (44.3)	1,688 (41.8)	2,354 (58.2)	
Short duration	1,913 (21.0)	994 (52.0)	919 (48.0)	

^*^p < 0.05.

**Figure 1:**
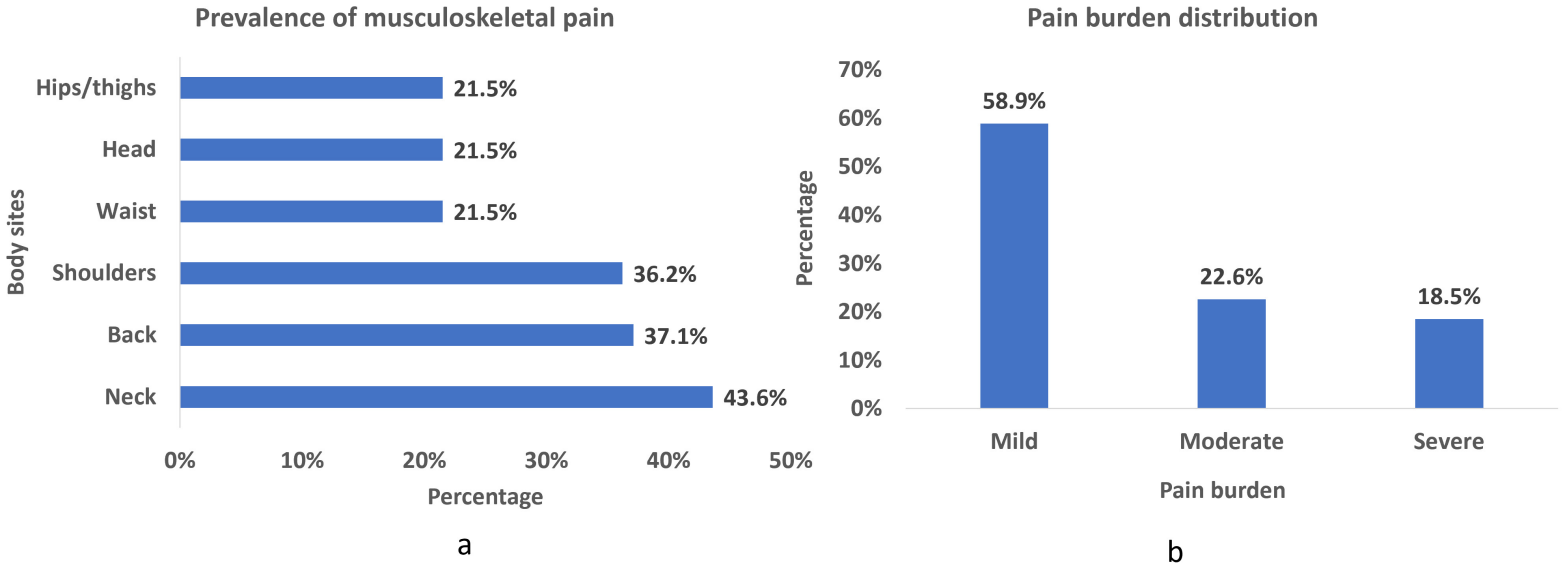
Prevalence of musculoskeletal pain. **(a)** prevalence of musculoskeletal pain by body site; **(b)** prevalence of musculoskeletal pain by severity.

MSP was highly prevalent, with 5,355 students (58.7%) reporting symptoms in at least one body site. As shown in Figure 1, the most frequently affected regions were the neck (43.6%), back (37.1%), and shoulders (36.2%), while waist (21.5%), head (21.5%), and hip/thigh (21.5%) pain were less common. The Pain Burden revealed that 22.6% of students experienced moderate pain and 18.5% experienced severe pain, indicating that MSP was not only widespread but also of considerable intensity.

Significant gender differences were observed, with more female students reporting MSP compared to males (67.8 vs. 51.4%, *p* < 0.001). Prevalence also varied by grade and major, with Ph.D. candidates reporting the highest prevalence (64.8%). Students in humanities reported higher pain prevalence (63.4%), followed by students in medicine (60.0%) and engineering (56.3%). Moreover, students with pain were older and had lower BMI compared to those without pain.

### 3.2 Lifestyle behaviors in university students

Lifestyle behaviors varied across domains. Physical activity levels were generally insufficient, with 33.4% students were classified as low physical activity (< 600 MET/week). Dietary habits were moderate in most students (55.3%), but irregular meal patterns (53%) and frequent consumption of sugar-sweetened beverages (≥3 days/week, 38%) were common. Only 34% students reported eating breakfast regularly, and fewer than half (47%) consumed three meals per day.

Sedentary behavior was moderate in the majority of students, with nearly half reporting >6 h of sitting per day and over half (52%) reporting continuous sitting for >1 h. Sleep emerged as the weakest lifestyle domain, with only 5.1% of students classified as having good sleep quality. Late bedtimes were widespread, with more than 8,000 students reporting sleep onset after 23:00, despite most achieving ≥7 h of sleep. Screen use was also problematic, with one third reporting >6 h of daily exposure and the majority exceeding 3 h per day.

**Figure 2:**
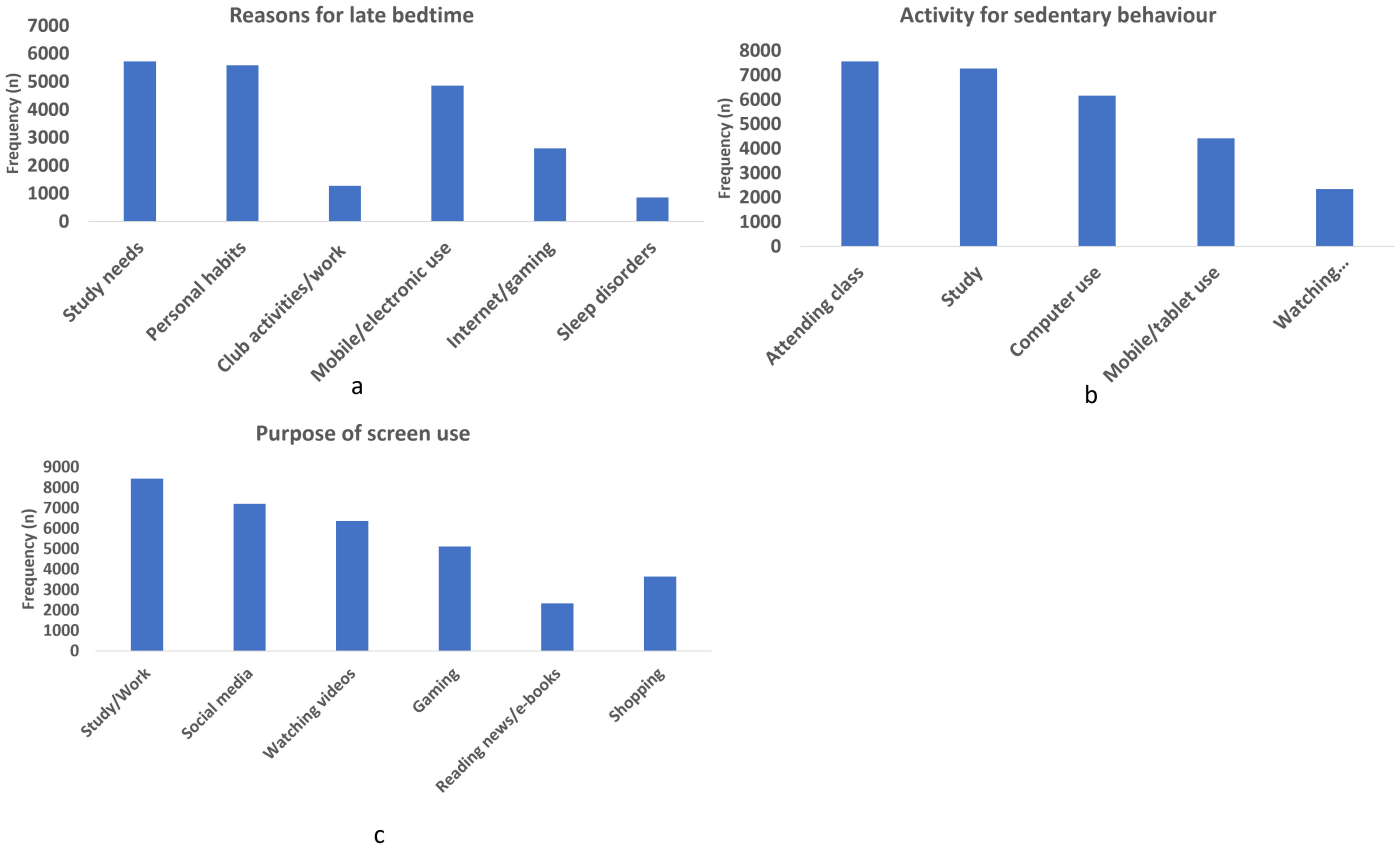
Behavioral drivers of late bedtime, sedentary activity, and screen use among university students. **(a**) Reasons for late bedtime; **(b)** activities contributing to sedentary behavior; **(c)** purposes of screen use.

In addition to lifestyle domains, behavioral drivers of late bedtime, sedentary activity, and screen use were examined. The most frequently reported reasons for late bedtime were academic and habitual, with study needs and personal habits being most common, followed by student union/work activities (Figure 2). Digital distractions also contributed, including mobile/electronic use and internet/gaming. Sleep disorders were reported by fewer than 1,000 students. Sedentary activity was primarily driven by academic routines, with attending class and study as leading causes, followed by computer use, mobile/tablet use, and watching TV/movies. Screen use was dominated by work/study, social media, and watching videos, with gaming, reading news/e-books, and shopping less frequent.

### 3.3 Risk factors for MSP in university students

Univariate and multivariate logistic regression analyses were performed to identify risk factors for MSP. Univariable logistic regression analyses indicated that age, gender, grade, major, BMI, physical activity, diet, sedentary behavior, sleep, and screen use were all potential risk factors for MSP.

Multivariable logistic regression (Table 2) confirmed that several factors remained independently associated with MSP prevalence after adjustment. Female gender was a strong predictor, with nearly double the odds of reporting pain compared to males (*OR* = 1.99, 95% CI [1.80, 2.19], *p* < 0.001). Poor sleep quality was the most influential lifestyle factor, with students reporting poor sleep having more than three times the odds of pain compared to those with good sleep (*OR* = 2.81, 95% CI [2.26, 3.49], *p* < 0.001). Poor diet (*OR* = 1.83, 95% CI [1.59, 2.10], *p* < 0.001), high sedentary risk (*OR* = 1.33, 95% CI [1.10, 1.60], *p* = 0.03), and long screen duration (*OR* = 1.49, 95% CI [1.31, 1.69], *p* < 0.001) were also significant independent predictors. Low physical activity increased the odds of pain prevalence compared with high activity (*OR* = 1.14, 95% CI [1.02, 1.27], *p* = 0.020), while moderate activity was not significant.

**Table 2:** Logistic regression analysis of risk factors for musculoskeletal pain.

Predictor	Univariable analysis (*p-*value)	Multivariable analysis (*p-*value)	OR	95% CI
Age	< 0.001^*^	0.954		
Gender	< 0.001^*^	< 0.001^*^	1.985	[1.797, 2.192]
BMI	0.001^*^	0.807		
Grade	< 0.001^*^	0.113		
Major	< 0.001^*^	0.687		
Lifestyle				
Physical activity				
Low	< 0.001^*^	0.020^*^	1.138	[1.021, 1.269]
Moderate	< 0.001^*^	0.244		
High	Ref	Ref		
Diet				
Poor	< 0.001^*^	< 0.001^*^	1.828	[1.589, 2.103]
Moderate	< 0.001^*^	< 0.001^*^	1.464	[1.304, 1.644]
Good	Ref	Ref		
Sedentary behavior				
High risk	< 0.001^*^	0.003^*^	1.324	[1.098, 1.596]
Moderate risk	< 0.001^*^	< 0.001^*^	1.257	[1.143, 1.382]
Low risk	Ref	Ref		
Sleep				
Poor	< 0.001^*^	< 0.001^*^	2.808	[2.260, 3.490]
Moderate	< 0.001^*^	< 0.001^*^	2.013	[1.632, 2.481]
Good	Ref	Ref		
Screen use				
Long duration	< 0.001^*^	< 0.001^*^	1.494	[1.314, 1.699]
Moderate duration	< 0.001^*^	< 0.001^*^	1.288	[1.148, 1.445]
Short duration	Ref	Ref		

Univariable *p*-values represent crude associations.

Multivariable *p*-values represent adjusted associations for each variable in the regression model.

Odds ratios and 95% confidence intervals are reported for individual categories relative to the reference group. Ref = reference category.

^*^p < 0.05.

### 3.4 Risk factors for pain burden

Multivariable ordinal logistic regression was used to identify independent predictors of MSP burden among students reporting pain. Demographic variables such as age, grade, and major were tested but showed no significant associations and were therefore excluded from the final model.

As summarized in Table 3, female gender was significantly associated with higher pain burden (*OR* = 1.90, 95% CI [1.73, 2.08], *p* < 0.001). Lifestyle factors were also independently associated with pain burden. Poor sleep quality emerged as the strongest predictor, with students reporting poor sleep having more than three times the odds of higher burden compared to those with good sleep (*OR* = 3.72, 95% CI [2.86, 4.83], *p* < 0.001). Moderate sleep quality also increased the odds of higher burden (*OR* = 2.44, 95% CI [1.88, 3.15], *p* < 0.001). Poor diet (*OR* = 1.90, 95% CI [1.66, 2.18], *p* < 0.001), moderate diet (*OR* = 1.32, 95% CI [1.17, 1.49], *p* < 0.001), high sedentary risk (*OR* = 1.68, 95% CI [1.42, 1.99], *p* < 0.001), moderate sedentary risk (*OR* = 1.27, 95% CI [1.16, 1.40], *p* < 0.001), and long screen use (*OR* = 1.37, 95% CI [1.20, 1.55], *p* < 0.001) were all significant predictors. Moderate screen use was also associated with higher burden, though with a smaller effect (*OR* = 1.13, 95% CI [1.01, 1.27], *p* = 0.042). Low physical activity was independently associated with higher pain burden compared with high activity (*OR* = 1.04, 95% CI [1.02, 1.15], *p* = 0.020), whereas moderate activity was not significant.

**Table 3:** Logistic regression analysis of risk factors for musculoskeletal pain burden.

Predictor	Multivariable analysis (*p-*value)	OR	95% CI
Gender	< 0.001^*^	1.896	[1.726, 2.083]
Lifestyle			
Physical activity			
Low	0.031^*^	1.042	[1.021, 1.154]
Moderate	0.742		
High	Ref		
Diet			
Poor	< 0.001^*^	1.904	[1.660, 2.184]
Moderate	< 0.001^*^	1.322	[1.173, 1.490]
Good	Ref		
Sedentary behavior			
High risk	< 0.001^*^	1.677	[1.417, 1.985]
Moderate risk	< 0.001^*^	1.271	[1.157, 1.397]
Low risk	Ref		
Sleep			
Poor	< 0.001^*^	3.718	[2.858, 4.832]
Moderate	< 0.001^*^	2.435	[1.883, 3.148]
Good	Ref		
Screen use			
Long duration	< 0.001^*^	1.365	[1.204, 1.547]
Moderate duration	0.042^*^	1.129	[1.005, 1.267]
Short duration	Ref		

Multivariable *p*-values represent adjusted associations for each variable in the ordinal logistic regression model.

Odds ratios and 95% confidence intervals are reported for individual categories relative to the reference group.

Ref = reference category.

^*^p < 0.05.

## 4 Discussion

This study revealed that MSP is highly prevalent among university students, affecting nearly six in 10 respondents. Lifestyle behaviors were generally from poor to moderate dependent on domains, and students with pain consistently demonstrated less favorable profiles. Regression analyses confirmed that poor sleep, poor diet, high sedentary risk, low physical activity, and long screen use duration were independent risk factors of pain, highlighting the close interconnection between lifestyle risks and pain.

### 4.1 Prevalence of pain and pain burden

Pain prevalence in this cohort was strikingly high, with 58.7% of students reporting symptoms in at least one body site. The neck, back, and shoulders were the most commonly affected regions, consistent with international studies reporting MSP rates of 50%−88% among university populations (26–28). Analysis of pain burden showed that more than 40% of students experienced moderate to severe pain, which underscores that pain is not only widespread but also of considerable intensity. A previous study conducted on Chinese students also indicated high prevalence of neck pain (44.4%) and low back pain (30.6%) with moderate severity (29). Moreover, our findings align with studies noting that prolonged sedentary study and increased digital exposure have exacerbated musculoskeletal complaints among students (12, 30). Importantly, our study extends existing literature by quantifying both prevalence and burden, showing that lifestyle risks contribute differently to pain occurrence and severity. This distinction adds novelty compared with earlier research that primarily focused on prevalence alone.

### 4.2 Demographic and lifestyle factors on MSP

This study identified multiple demographic and lifestyle factors associated with MSP among university students. Consistent with previous literature, female gender was strongly associated with both pain prevalence and burden, underscoring gender differences in musculoskeletal vulnerability. Women generally have greater pain sensitivity, influenced by hormonal fluctuations, psychosocial stress, and coping strategies (31, 32).

Apart from lifestyle risk factors on prevalence of pain we also investigate lifestyle risk factors on pain burden which reflect the severity of pain. Pain burden analysis revealed that lifestyle factors not only influenced the likelihood of pain but also its severity. Predictors of pain prevalence and pain burden overlapped but differed in magnitude and significance.

#### 4.2.1 Physical activity and pain

Insufficient physical activity was common, with 33.4% students classified as low physical activity. The WHO recommend at least 150 min of moderate-intensity/week physical activity, which is equal to the physical activity level in moderate group (33). It shows that only 60% students meet the WHO bottom line. Low physical activity was independently associated with both pain prevalence and burden compared with high activity levels. This indicates that insufficient activity not only increases the likelihood of developing MSP but also exacerbates its severity once present. No difference was found in pain prevalence and pain burden between the students with moderate activity levels and those with high level, suggesting once arriving at the physical level of WHO recommendation, the protective effect is significant. Prior research also shows that inadequate physical activity contributes to neck and shoulder pain among student populations (11, 12, 34).

#### 4.2.2 Diet and pain

Dietary behavior was moderate overall, but poor dietary patterns were strongly associated with pain prevalence and pain burden. The influence of poor diet on pain burden was more pronounced (pain prevalence *OR* = 1.82 and pain burden *OR* = 1.90), indicating that poor nutrition promotes systemic inflammation and metabolic stress, exacerbating musculoskeletal discomfort (35). We found that irregular meals and high consumption of sugar-sweetened beverages were particularly common among students with pain which is consistent with the results from other study (36). Recent evidence also confirm that poor diet quality is a significant risk factor for pain (37, 38). On the contrary, balanced diets rich in fruits, vegetables, and whole grains may provide anti-inflammatory benefits and improve recovery, highlighting diet as a critical domain for intervention.

#### 4.2.3 Sleep and pain

Sleep emerged as the weakest lifestyle domain and the strongest risk factors in both pain prevalence and pain burden. While its impact on pain burden was substantially stronger (*OR* = 3.72 vs. *OR* = 2.81 for prevalence), suggesting that poor sleep not only increases the risk of pain but intensifies its severity. Students with poor sleep behavior had nearly threefold higher odds of pain, reflecting the significant influence of sleep on pain perception and recovery. Late bedtimes and irregular schedules were widespread, despite adequate sleep duration in many cases which is common among university students (39). Other studies observed the association between sleep and MSP as well (40, 41). Prior studies confirm that poor sleep quality amplifies pain sensitivity through impaired neurobiological modulation and heightened inflammatory responses (42). Furthermore, our findings highlight the strong influence of academic demands and personal routines on sleep behavior among university students. All these findings suggest that sleep hygiene interventions may be among the most effective strategies for reducing pain in university populations.

#### 4.2.4 Sedentary behavior and pain

Sedentary behavior was moderate in most students, with nearly half reporting more than 6 h of sitting per day. Similar to our findings, a systematic review showed that the average university student reported engaging in sedentary behavior for about 7 h per day (43). The WHO has recommended to reduce sedentary behaviors across all age groups, although evidence was insufficient to quantify a sedentary behavior threshold (44). Our results indicated that high sedentary risk was independently associated with pain. It has been reported that prolonged sitting increases spinal pressure, reduces circulation, and promotes muscle fatigue (43, 44). Sedentary behavior was largely driven by classroom attendance and self-study, underscoring pressures of university life. Previous study also report that educational environments contribute significantly to postural strain and inactivity (45). Reviews of sedentary behavior across occupational and student populations confirm its strong association with MSP (13). Interventions encouraging movement breaks and ergonomic awareness may therefore help mitigate pain risk.

#### 4.2.5 Screen use and pain

Screen use was problematic, with one-third of students reporting more than 6 h of daily exposure. Poor screen use was significantly associated with pain, reflecting the combined effects of poor posture, repetitive strain, and sleep disruption. Similar findings have been reported in other cohorts, where excessive screen exposure was a major driver of musculoskeletal complaints (45). Long screen use duration was also risk factors to pain burden, but the influence on pain prevalence was more pronounce. Screen use was primarily functional (work/study), but social and entertainment purposes also accounted for substantial exposure. The overlap between screen use and sleep disruption, particularly via mobile devices, may exacerbate MSP through poor posture and circadian misalignment. Given the centrality of digital devices in academic life, ergonomic training and digital wellness education should be prioritized to reduce screen-related pain.

### 4.3 Public health implications

Our findings emphasize that MSP among university students is not only a clinical issue but also a lifestyle-related public health challenge. The high prevalence and intensity of pain, coupled with multiple lifestyle risks, call for integrated campus health strategies.

Importantly, our analysis highlights the need to distinguish between pain occurrence and pain burden. While prevalence reflects how widespread pain is, burden captures its severity and impact. Sleep quality and diet were consistently strong predictors across both models, indicating that poor sleep and poor nutrition not only pre-dispose students to pain but also intensify its severity. Our results show that low physical activity was independently associated with both pain prevalence and burden compared with high activity levels. This demonstrates that insufficient activity contributes to pain onset and also exacerbates symptom severity once pain develops.

These differences have practical implications. Interventions should not only aim to reduce the number of students experiencing pain but also mitigate the intensity of symptoms among those affected. Strategies that prioritize sleep hygiene, dietary improvements, and ergonomic screen use and promotion of physical activity may be particularly impactful in reducing both pain prevalence and burden.

By addressing both behavioral and structural determinants, universities can reduce pain burden, enhance student wellbeing, and support healthier long-term habits. Tailored approaches that consider gender-specific vulnerabilities will ensure interventions are both effective and equitable.

### 4.4 Limitations

A key strength of this study lies in its large sample size of over 9,000 students, which provides robust estimates of pain prevalence and lifestyle behaviors. The use of a composite pain burden allowed stratification by severity, offering a nuanced understanding beyond simple prevalence, while the multidomain assessment of lifestyle factors enabled comprehensive analysis of risk patterns.

Nevertheless, several limitations should be acknowledged. The cross-sectional design prevents causal inference, meaning that while lifestyle factors were associated with pain, temporal relationships cannot be established. Data were self-reported, raising the possibility of recall bias and social desirability bias, particularly for sensitive behaviors such as diet and sleep. The study was conducted at a single university, which may limit generalizability to other student populations with different cultural or academic contexts. Lifestyle domains were categorized into broad groups, which may not fully capture the complexity or interactions of behaviors.

In addition, although data were collected across different student majors, multivariable regression did not identify major as a significant independent predictor of musculoskeletal pain prevalence or burden. Gender was the only demographic factor retained in the final models. This suggests that discipline-specific differences may not exert a direct statistical effect once lifestyle factors are accounted for. However, academic contexts likely shape exposure to risk behaviors—for example, prolonged sedentary time in laboratory-based majors, posture strain in arts majors, sleep disruption in medical majors, or overuse injuries in sports majors. Because these contextual influences were not captured as significant in our adjusted models, caution is warranted in generalizing across disciplines. Future research should stratify analyses by major and explore contextual demands more deeply to determine whether tailored interventions are needed for specific student groups.

Despite these limitations, the findings provide valuable evidence on the interplay between pain and lifestyle among university students and highlight the need for future longitudinal, multi-site research to confirm and extend these results.

## 5 Conclusion

This study provides robust evidence on the prevalence and burden of MSP among university students and highlights the role of lifestyle behaviors in shaping pain outcomes. Using a large sample of over 9,000 students and a composite measure of pain burden, we demonstrated that insufficient physical activity, poor sleep quality, poor diet, high sedentary risk, and prolonged screen use were all significant contributors to MSP. Among these, poor sleep and poor diet emerged as the strongest predictors of pain burden, underscoring their influence on symptom severity. Gender was the only demographic factor independently associated with MSP, with female students reporting higher prevalence and burden. Although academic major was not statistically significant in multivariable models, contextual differences across disciplines may still shape exposure to risk behaviors, suggesting the need for tailored interventions in future research.

These findings emphasize that MSP among university students is not only a clinical issue but also a lifestyle-related public health challenge. Interventions should prioritize sleep hygiene, dietary improvements, reduction of sedentary behavior, ergonomic screen use, and promotion of sustained physical activity. Addressing both behavioral and structural determinants can reduce pain prevalence and mitigate burden, thereby enhancing student wellbeing and academic performance. Future longitudinal, multi-site studies should explore discipline-specific contexts and integrate psychological, physiological, and socioeconomic factors to provide a more comprehensive understanding of MSP risk.

## Data Availability

The datasets presented in this article are not readily available because the dataset generated and analyzed during the current study is not publicly available due to restrictions imposed by the Ethics Review Committee of the West China School of Public Health, Sichuan University. Specifically, the dataset contains sensitive personal health and lifestyle information from university students, and sharing is limited to protect participant confidentiality. Data access is therefore restricted to the research team and may be provided in anonymized form upon reasonable request, subject to ethical approval and institutional data-sharing agreements. Requests to access the datasets should be directed to woshiliyan2013@gmail.com.
